# Combined Endonasal-Transcervical Approach to a Metastatic Parapharyngeal Space Papillary Thyroid Carcinoma

**DOI:** 10.7759/cureus.285

**Published:** 2015-07-14

**Authors:** Arnau Benet, Julio Plata Bello, Ivan El-Sayed

**Affiliations:** 1 Department of Otolaryngology-Head and Neck surgery. Department of Neurosurgery, University of California San Francisco; 2 Neurosurgery, Hospital Universitario de Canarias; 3 Otolaryngology Head and Neck Surgery, University of California San Francisco

**Keywords:** parapharyngeal space, endoscopic endonasal, transcervical, papillary thyroid carcinoma, transmaxillary-transpterygoid

## Abstract

*Purpose*: Although papillary thyroid carcinoma metastases to the parapharyngeal space are rare, the high amount of fat tissue allows tumors to grow clinically undetectable until they invade most of the parapharyngeal space. We describe for the first time a combined endonasal and transcervical approach for a parapharyngeal metastasis from a papillary thyroid carcinoma.

*Materials and Methods*: A 51-year-old male with a previous history of papillary thyroid carcinoma presented with left ear fullness and left-sided facial numbness. Imaging revealed a 4x3 cm pre-styloid parapharyngeal space mass invading the foramen ovale and extending below the palate. Needle biopsy confirmed metastatic papillary thyroid carcinoma.

*Results*: The lesion was resected with a combined endoscopic endonasal and transcervical approach. Postoperative MRI revealed gross total resection, and the patient recovered from his symptoms.

*Conclusion*: This novel approach provides access to pre-styloid parapharyngeal tumors with superior extension to the skull base, avoiding more extensive traditional open approaches.

## Introduction

The parapharyngeal space (PS) is the medial compartment of the infratemporal fossa. It extends from the skull base to the hyoid bone and is limited by the pterygomandibular raphe anteriorly, by the inner fascia of the masticatory space and the deep lobe of the parotid gland laterally, and by the buccopharyngeal and alar fasciae medially. The posterior limit of the PS is generally regarded to extend to the posterior fascia of the carotid sheath. The PS is divided into pre- and retro-styloid compartments by the tensor-vascular-styloid (TVS) fascia. The pre-styloid compartment contains fatty tissue, the salivary gland, the mandibular division of nerve V, and the internal maxillary and ascending pharyngeal arteries. The retro-styloid compartment contains the carotid artery, jugular vein, nerves IX-XII, sympathetic chain, and lymphatic nodes. Rouviere (1938) identified a consistent lymphatic trunk connecting the superior limit of the lateral thyroid lobe to the retropharyngeal lymphatic nodes [1]. Moreover, the retropharyngeal lymphatic nodes can connect to the parapharyngeal space through a dehiscence of the superior constrictor muscle fascia.

Lesions in the PS affecting the skull base with risk of intracranial invasion and spread to vital neurovascular structures require definitive intervention [2]. The PS has been surgically accessed using the transcervical, transoral, mandibular swing, transparotid, transmastoid, and infratemporal fossa approaches and their combinations [3-4], but the optimal surgical approach depends on the location of the lesion within the PS. In this sense, tumors that arise in most superior and medial areas (e.g. pterygopalatine fossa), such as in this case report, present a limited access with those approaches. Because of that, other approaches must be employed for managing these lesions. With this in mind, an endoscopic transnasal approach has been recently proposed as a feasible way for resecting tumors in the upper PS regions [5-7].

We describe a case of papillary thyroid carcinoma metastasis to the pre-styloid PS involving the skull base treated surgically using a combined endonasal transpterygoid approach and transcervical approach simultaneously. To our knowledge, this is the first reported case of a combined endoscopic endonasal approach (EEA) and a transcervical (TC) approach to the pre-styloid compartment of the PS.

## Case presentation

Informed patient consent was obtained prior to all treatments.

**Figure 1:**
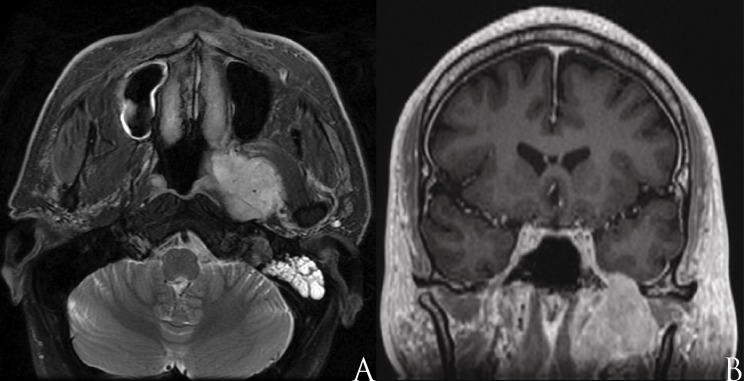
Preoperative magnetic resonance imaging. Axial T2-weighted magnetic resonance (MR) with gadolinium contrast reveals a mass located in the left parapharyngeal space (A). The lesion invades the pterygopalatine fossa and displaces the lateral pterygoid muscle anteriorly. (B) Coronal T1 post-gadolinium MR reveals the mass to extend to the intracranial space through foramen ovale without intradural or cavernous sinus invasion.

A 51-year-old male with a previous history of metastatic papillary thyroid carcinoma presented with lower facial numbness and a fullness sensation on the left ear for four months. The patient’s medical history includes a total thyroidectomy and radiotherapy with Iodine-131 six years prior. Three years later, he developed metastatic disease to the left neck and internal jugular vein that was treated with a left neck dissection, including jugular vein resection followed by postoperative radiotherapy to 6600 cGy. Three more years later, he subsequently developed a left serous effusion and marked numbness in the left V3 territory. A myringotomy with fluid evacuation as well as magnetic resonance imaging (MRI) with gadolinium of the head and neck were performed. The MRI revealed a 4 x 3.3 cm highly vascularized tumor in the left pre-styloid PS with foramen ovale invasion (Figure 1). A fine needle biopsy confirmed a papillary thyroid carcinoma metastasis. A preoperative endovascular embolization of the tumor feeders, including the left distal internal maxillary, accessory meningeal, and pharyngeal branch of the ascending pharyngeal artery, was performed two days prior to surgery.

Bearing in mind the prior treatment given to neck and the close relationship of the tumor with the foramen ovale, and considering the complexity of approaching this location only by a TC access, a combined TC and EEA approach was considered. 

**Figure 2:**
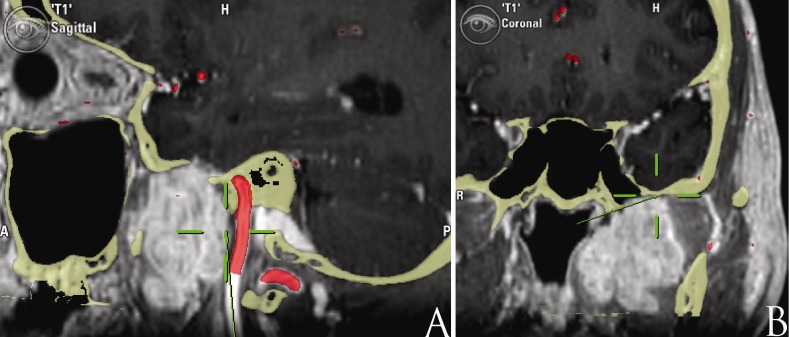
Computed tomography overlay T1-weighted MRI and MRA scan. The arterial vasculature is labeled (red). The image guidance probe (green line) indicates that the carotid artery was accessed and detached from the tumor from the transcervical approach (A) and that foramen ovale was approached and the superior limit of the tumor was accessed through the endoscopic endonasal route (B).

A simultaneous, transmaxillary-transpterygoid endoscopic EEA and TC approach was performed on the left side of the neck. The standard transcervical approach was performed first as described in the literature [3]. The styloid process was resected and the retro-styloid compartment was identified. The carotid artery and lower cranial nerves were identified and dissected to the skull base away from the tumor (Figure 2A). The vessel was protected with cottonoid pledgets. The tumor was dissected free along its anterior, inferior, and lateral margins and detached from the pterygoid muscle and medial constrictor muscle.

Subsequently, the superior and medial margins were approached through the EEA. The left nasal cavity was accessed through the left nostril and the middle turbinate was removed. The medial maxillary wall was removed, along with the piriform process and 5 mm of the anterior maxillary wall to access the maxillary sinus [8]. Next, the posterior and lateral walls of the maxillary sinus were removed to expose the pterygopalatine fossa. The internal maxillary artery was identified laterally and ligated with endoscopic clips. The transpterygoid approach was carried out to dissect the tumor from the Eustachian tube [9], expose the foramen ovale, and delineate the tumor involvement at the skull base (Figure 2B). At this point, the foramen ovale and rotundum were dissected preserving V2 and V3. The dura exposed was in direct contact to the tumor, but no signs of an invasion were observed. The combined approach provided a 360-degree access to the tumor, allowing the direct control of the neurovascular structures and a safe PS dissection.  

**Figure 3:**
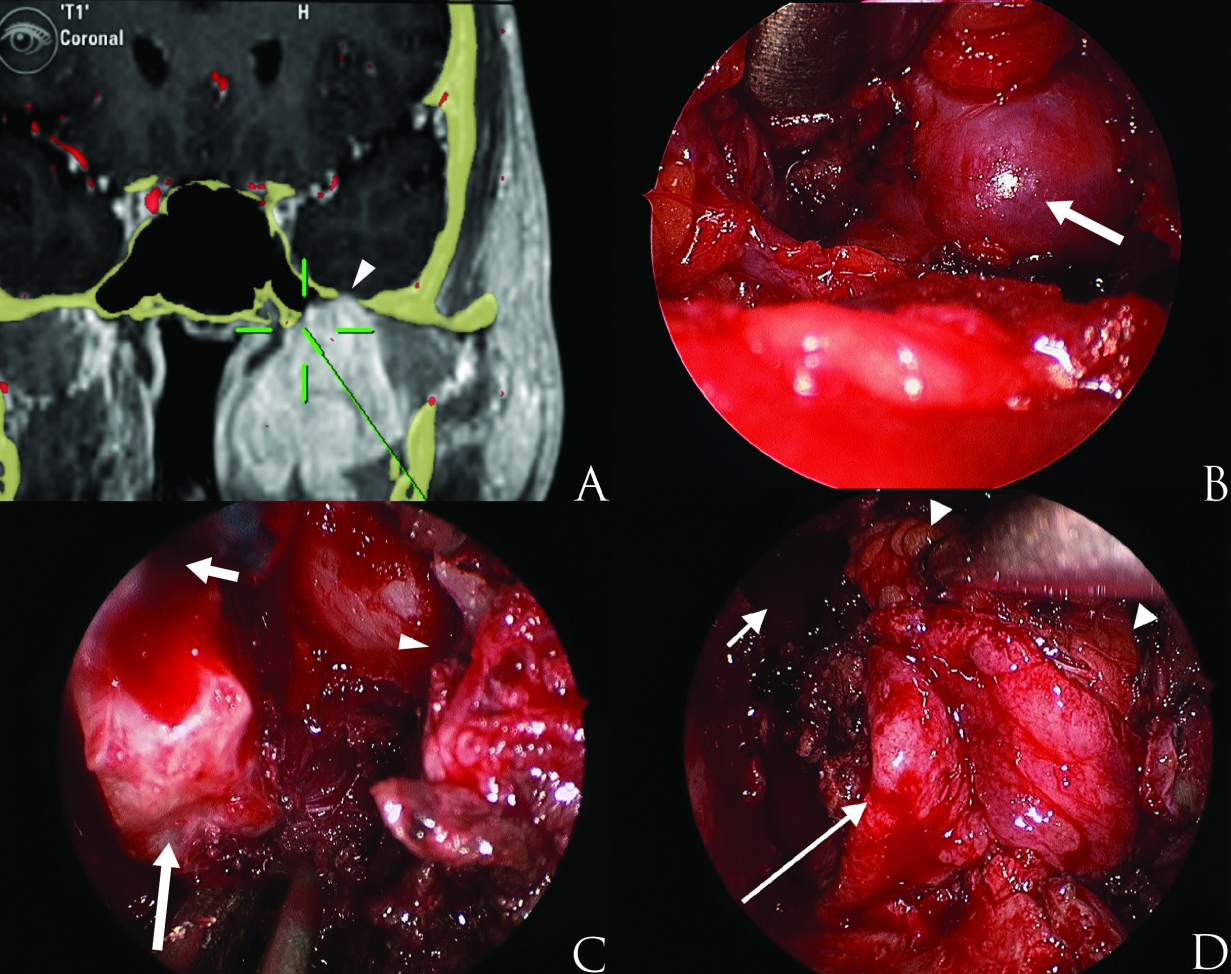
Surgical dissection of the parapharyngeal tumor. (A) The image guidance probe is passed from neck after tumor is resected. The tumor eroded the foramen ovale (arrowhead) and the lateral recess of the sphenoid sinus, which was the most medial region accessed through the transcervical approach. (B) The inferior edge of the tumor is identified through the neck (arrow) but access to adjacent structures is limited superiorly. (C) The lateral pterygoid plate has been resected endonasaly (long arrow) just below the opening to the lateral recess of the sphenoid sinus (short arrow). A rim of bone with tumor extending to the foramen ovale is identified (arrowhead). (D) A pedicled flap is elevated from the nasal cavity floor and rotated over the medial defect (long arrow). A buccal fat pad flap (two arrowheads) is elevated laterally and mobilized to the lateral edge of the sphenoid sinus (short arrow).

The wound defect was closed using vascularized buccal fat and a pedicled mucosal flap harvested from the floor of the nasal fossa (Figure 3). Postoperative MRI revealed a gross total resection of the mass. The patient was discharged from the hospital on postoperative day five without significant cranial nerve dysfunction or wound complication. An abbreviated CyberKnife radiation treatment was given to the dura at the foramen ovale. One year after the operation, the patient recovered sensation over the face and had no left ear symptoms.

## Discussion

Surgical resection is the most effective treatment for PS tumors [10]. Superiorly located PS lesions are challenging to resect and require an extended approach to avoid injury to adjacent retro-styloid neurovascular structures. When cranial base structures are involved, the approaches that are normally used may not be sufficient to achieve a complete resection.

The PS has been surgically accessed using the transcervical, transoral, mandibulotomy or mandibular swing, transparotid, transmastoid, and infratemporal fossa approaches and their combinations [4, 11-12]. Although no strict guidelines exist, the approach is generally selected based on the location of the lesion, its size, and the tumor biology. The transoral approach is proposed to resect small lesions (< 3 cm) involving the oropharynx that displace the tonsillar region medially. Mandibular swing and mandibulotomy provide excellent access to large lesions located in the inferior half of the pre-styloid space involving the pterygomandibular raphe, pterygomaxillary fissure, and buccopharyngeal fascia, and they can aid access to superior lesions as well. Adverse outcomes include scar in the lower lip, paralysis of the mandibular branch of the VII nerve, malocclusion, non-union of the mandible, and temporal mandibular joint arthrosis. The transparotid approach provides access to the lateral aspect of both pre- and retro-styloid compartments of the PS; however, access to the most cranial and caudal regions of the PS is limited [4]. Moreover, dissection of the facial nerve increases the risk of paralysis. The subtemporal preauricular infratemporal approach provides excellent access to the PS as well as the intracranial space and subtemporal dura matter with direct exposure of the pterygomaxillary fissure, foramen spinosum and ovale, and the carotid sheath while the infratemporal-posterior fossa approach provides access to the jugular foramen and bulb [11]. These approaches require extensive tissue dissection and harbor potential facial nerve paralysis, internal maxillary and superficial temporal artery hemorrhage, malocclusion, and bacterial meningitis.

The TC approach offers direct access to the caudal PS. It is particularly suited to pre-styloid tumors and offers an optimal caudal-cranial dissection plane between the tumor and the TVS, which ease posterior detachment under direct visualization of the carotid artery (Figure 3B). However, the TC approach gives limited access to the cranial base and pterygopalatine fossa, which can be safely approached endoscopically.

In the case reported here, we have employed a combined approach to achieving a complete resection of left pre-styloid PS tumor with foramen ovale invasion. The TC approach allowed exposure of the inferior two-thirds of the tumor. The EEA provided direct exposure of the pterygopalatine and infratemporal fossae and skull base [9] with an excellent view of the infraorbital nerve, cranial nerve V3, sphenoid sinus, Eustachian tube, internal maxillary artery, and foramen lacerum at the skull base (Figure 3C). This allowed tumor dissection at the foramen ovale under direct view. After the combined approach, the tumor was dissected in 360-degrees with progressive detachment from the surrounding critical structures always under direct view; thus, a complete resection of the tumor could be achieved. In the face of metastatic disease, the goal of the surgery was for symptom reduction and to prevent the imminent intracranial spread of disease. The least morbid approach was sought to alleviate symptoms. The patient was placed on ongoing systemic therapy with Phase I trials shortly after treatment.

The combination of the TC approach and the EEA was considered due to the extension of the lesion. As it was discussed above, the TC approach alone would not allow having a complete control during the dissection of the tumor medially. On the other hand, the EEA approach alone is not adequate to resect a large papillary thyroid carcinoma metastasis invading the PS for two reasons: first, the nasal corridor is limited inferiorly, and second, identification of the carotid artery is difficult when the tumor is approached from medially alone since there are few landmarks to the carotid artery. Therefore, although the TC approach could also be combined with other approaches described above (e.g. transmandibular), the combination of TC and EEA approaches described in the present case may allow achieving a larger resection safely with control of neurovascular structures in the management of the PS tumors with cranial base involvement.

## Conclusions

A combined EEA and TC approach is a good strategy for the surgical removal of large tumors of the PS with marked medial invasion. This strategy provides a 360-degree dissection of the pre-styloid PS tumors, allowing safe dissection of delicate structures of the pterygopalatine fossa, skull base, and the inferior aspect of the PS under direct visualization. 

## References

[1] Rouviere H (1938). Anatomy of the Human Lymphatic System. Anat. Hum. Lymphat. Syst. Brothers.

[2] Wang XL, Xu ZG, Wu YH, Liu SY, Yu Y (2012). Surgical management of parapharyngeal lymph node metastasis of thyroid carcinoma: a retrospective study of 25 patients. Chin Med J (Engl).

[3] Chang SS, Goldenberg D, Koch WM (2012). Transcervical approach to benign parapharyngeal space tumors. Ann Otol Rhinol Laryngol.

[4] Bozza F, Vigili MG, Ruscito P, Marzetti A, Marzetti F (2009). Surgical management of parapharyngeal space tumours: results of 10-year follow-up. Acta Otorhinolaryngol Ital.

[5] Battaglia P, Turri-Zanoni M, Dallan I, Gallo S, Sica E, Padoan G, Castelnuovo P (2014). Endoscopic endonasal transpterygoid transmaxillary approach to the infratemporal and upper parapharyngeal tumors. Otolaryngol Head Neck Surg.

[6] Dallan I, Lenzi R, Bignami M, Battaglia P, Sellari-Franceschini S, Muscatello L, Seccia V, Castelnuovo P, Tschabitscher M (2010). Endoscopic transnasal anatomy of the infratemporal fossa and upper parapharyngeal regions: correlations with traditional perspectives and surgical implications. Minim Invasive Neurosurg.

[7] Van Rompaey J, Suruliraj A, Carrau R, Panizza B, Solares CA (2013). Access to the parapharyngeal space: an anatomical study comparing the endoscopic and open approaches. Laryngoscope.

[8] El-Sayed I, Pletcher S, Russell M, McDermott M, Parsa A (2011). Endoscopic anterior maxillotomy: infratemporal fossa via transnasal approach. Laryngoscope.

[9] Falcon RT, Rivera-Serrano CM, Miranda JF, Prevedello DM, Snyderman CH, Kassam AB, Carrau RL (2011). Endoscopic endonasal dissection of the infratemporal fossa: Anatomic relationships and importance of eustachian tube in the endoscopic skull base surgery. Laryngoscope.

[10] Desuter G, Lonneux M, Plouin-Gaudon I, Jamar F, Coche E, Weynand B, Rahier J, Grégoire V, Andry G, Hamoir M (2004). Parapharyngeal metastases from thyroid cancer. Eur J Surg Oncol.

[11] Kanzaki S, Nameki Nameki (2008). Standardised method of selecting surgical approaches to benign parapharyngeal space tumours, based on pre-operative images. J Laryngol Otol.

[12] Riffat F, Dwivedi RC, Palme C, Fish B, Jani P (2014). A systematic review of 1143 parapharyngeal space tumors reported over 20 years. Oral Oncol.

